# 1218. Retrospective Audit of Vancomycin Utilization after Initiation of Methicillin Resistant Staphylococcus aureus (MRSA) Screening in Hematology and Bone Marrow Transplant (BMT) Patients.

**DOI:** 10.1093/ofid/ofad500.1058

**Published:** 2023-11-27

**Authors:** Neeraja Swaminathan, Kelsie Cowman, Margaret E McCort, Priya Nori, Terrence D McSweeney, Yi Guo, Rachel Bartash

**Affiliations:** University of Utah, Salt Lake City, Utah; Montefiore Medical Center, Bronx, New York; Montefiore Medical Center / Albert Einstein College of Medicine, Bronx, New York; Montefiore Health System, Bronx, NY; Montefiore Medical Center, Bronx, New York; Montefiore Medical Center, Bronx, New York; Montefiore Medical Center, Bronx, New York

## Abstract

**Background:**

Inappropriate vancomycin use in patients with febrile neutropenia (FN) can contribute to emergence of resistant organisms, acute kidney injury, and infusion related adverse effects. Methicillin resistant Staphylococcus aureus (MRSA) nares Polymerase Chain Reaction (PCR) testing has a high negative predictive value for invasive MRSA infections and therefore, offers a rapid tool to help avoid or de-escalate anti-MRSA therapy. In this study, we aimed to assess the impact of MRSA nares PCR on the utilization of vancomycin in a hematology/bone marrow transplant (BMT) unit.

**Methods:**

Our hematology/BMT unit is a closed 32-bed unit with patients undergoing BMT, receiving care for complications related to BMT or undergoing chemotherapy for hematologic malignancies. Nasal MRSA PCR testing was added to our institutional FN treatment protocol in September 2021 to help guide antibiotic use for this patient population. As part of our antimicrobial stewardship program’s audit and feedback and antimicrobial utilization monitoring, we measured the use of intravenous (IV) vancomycin on the unit before (August 2020-August 2021) and after (October 2021-March 2023) updating our guidelines to include MRSA PCR. Vancomycin utilization, which was obtained from data submitted to National Healthcare Safety Network (NHSN), was measured using unit-level days of therapy (DOT) per 1000 days present. The incidence density rates were compared using the NHSN statistics calculator.

**Results:**

Mean IV vancomycin use pre-MSRA PCR implementation was 91.27 DOT per 1000 days present, compared to 52.69 DOT per 1000 days present post MRSA PCR implementation (p < 0.001) (Figure 1). The monthly trend of vancomycin DOT per 1000 days present is shown in Figure 2.

**Figure:**
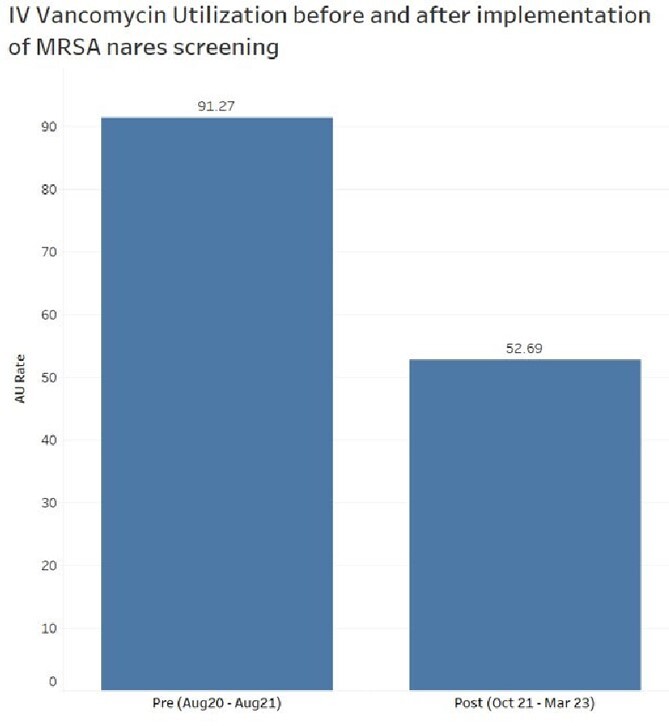

**Figure:**
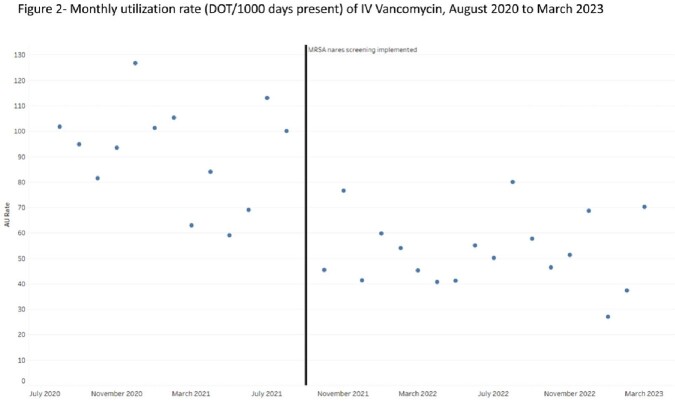

**Conclusion:**

Vancomycin utilization significantly decreased after the implementation of MRSA nares PCR as a part of the neutropenic fever workup. This study highlights its utility as a tool to guide therapy specifically in immunocompromised patients with significant antibiotic exposure.

**Disclosures:**

**All Authors**: No reported disclosures

